# The degree of cortisol secretion is associated with diabetes mellitus and hypertension in patients with nonfunctioning adrenal tumors

**DOI:** 10.1186/s12933-023-01836-1

**Published:** 2023-05-02

**Authors:** Vittoria Favero, Carmen Aresta, Chiara Parazzoli, Elisa Cairoli, Cristina Eller-Vainicher, Serena Palmieri, Antonio Stefano Salcuni, Maura Arosio, Luca Persani, Alfredo Scillitani, Valentina Morelli, Iacopo Chiodini

**Affiliations:** 1 Department of Medical Biotechnology and Translational Medicine, University of Milan, Milan, Italy; 2 Endocrinology Department & Lab of Endocrine and Metabolic Research, IRCCS - Istituto Auxologico Italiano, Milan, Italy; 3 Unit of Endocrinology, Fondazione IRCCS Cà Granda – Ospedale Maggiore Policlinico, Milan, Italy; 4 Unit of Endocrinology and Metabolism, University-Hospital S. Maria Della Misericordia, Udine, Italy; 5 Department of Clinical Sciences and Community Health, University of Milan, Milan, Italy; 6 Ospedale “Casa Sollievo della Sofferenza” IRCCS, San Giovanni Rotondo, FG Italy; 7 Unit of Endocrinology, Ospedale Niguarda Cà Granda, Milan, Italy

**Keywords:** Cortisol, 1-mg overnight dexamethasone suppression test, Nonfunctioning adrenal tumours, Hypocortisolism

## Abstract

**Background:**

Similarly to cortisol-secreting adrenal tumors, also non-functioning adrenal tumors (NFAT) may be associated with an increased cardiovascular risk. We assessed in NFAT patients: (i) the association between hypertension (HT), diabetes mellitus (DM), obesity (OB), dyslipidemia (DL) and cardiovascular events (CVE) and cortisol secretion; (ii) the cut-off of the cortisol secretion parameters for identifying NFAT patients with a worse cardiometabolic profile.

**Patients and methods:**

In 615 NFAT patients (with cortisol levels after 1 mg overnight dexamethasone suppression test, F-1mgDST < 1.8 µg/dL [50 nmol/L]) F-1mgDST and adrenocorticotroph hormone (ACTH) levels and data on HT, DM, OB, DL and CVEs prevalence were retrospectively collected.

**Results:**

HT, DM and HT *plus* DM were associated with F-1mgDST levels (area under the ROC curve: 0.588 ± 0.023, 0.610 ± 0.028, 0.611 ± 0.033, respectively, p < 0.001 for all comparisons) but not with ACTH. The cut-off for identifying patients with either HT or DM or HT *plus* DM was set at ≥ 1.2 µg/dL (33 nmol/L). As compared with patients with F-1mgDST < 1.2 µg/dL (n = 289), patients with F-1mgDST 1.2–1.79 µg/dL (33–49.4 nmol/L) (n = 326) had lower ACTH levels (17.7 ± 11.9 vs 15.3 ± 10.1 pg/mL, respectively, p = 0.008), older age (57.5 ± 12.3 vs 62.5 ± 10.9 years, respectively, p < 0.001), and higher prevalence of HT (38.1% vs 52.5% respectively p < 0.001), DM (13.1% vs 23.3%, respectively, p = 0.001), HT *plus* DM (8.3% vs 16.9%, respectively, p < 0.002) and CVE (3.2% vs 7.3%, respectively, p = 0.028). F-1mgDST 1.2–1.79 µg/dL was associated with either HT (odd ratio, OR, 1.55, 95% confidence interval, 95% CI 1.08–2.23, p = 0.018) or DM (OR 1.60, 95% CI 1.01–2.57, p = 0.045) after adjusting for age, gender, OB, DL, and DM (for HT) or HT (for DM), and with the presence of HT *plus* DM (OR 1.96, 95% CI 1.12–3.41, p = 0.018) after adjusting for age, gender, OB and DL.

**Conclusions:**

In NFAT patients, F-1mgDST 1.2–1.79 µg/dL seems to be associated with a higher prevalence of HT and DM and a worse cardiometabolic profile, even if the poor accuracy of these associations suggests caution in interpreting these results.

## Introduction

In recent years the management of patients with incidentally discovered adrenal masses (adrenal incidentalomas, AI) has become a topic of growing interest [1, 2]. The importance of AI in the clinical practice is due to the accumulating evidence that in up to 50% of AI patients may have a mild autonomous cortisol secretion (MACS), defined by the presence of hypercortisolism in the absence of the classic signs and symptoms of cortisol excess [2]. The interest in this condition is due firstly to the fact that the prevalence of this hidden cortisol excess in the adult population is not negligible, being estimated to reach 2% in adults over 60 years of age [3]. Secondly, MACS has been found to be associated with increased mortality, mainly due to a higher risk of cardiovascular events [4–9]. This is partially explained by the increased frequency in MACS patients of cardiovascular risk factors, diabetes mellitus (DM) and hypertension (HT) [10, 11]. A third argument is the improvement of DM and HT in MACS patients after adrenalectomy [12–15].

Interestingly, some recent data have suggested that there is an increased risk of cardiovascular events (CVE) even in patients with nonfunctioning adrenal tumors (NFAT) [16–19]. Accordingly, the mortality risk associated with the presence of NFAT seems to be comparable to that in AI patients with MACS [20], and adrenalectomy seems to be beneficial on HT and DM even in patients with NFAT [14]. Finally, a recent study by our group shows that a not negligible percentage of patients with AI without MACS who undergo unilateral adrenalectomy because of the size of the adenoma may experience a post-surgical hypocortisolism, which should not be expected in the absence of preoperative MACS [21]. In this study, cortisol after 1 mg overnight dexamethasone suppression test (F-1mgDST) levels measured before surgery with a cut-off set at 1.2 µg/dL (33 nmol/L)—and not 1.8 µg/dL (50 nmol/L) as suggested by current guidelines [1]—could completely rule out the risk of postoperative hypocortisolism. Moreover, although the small sample size did not allow us to draw firm conclusions, in this cohort AI patients with F-1mgDST < 1.2 µg/dL (33 nmol/L) tended to have a better metabolic profile than those with F-1mgDST ≥ 1.2 µg/dL [21]. As the occurrence of hypocortisolism after the removal of a unilateral adrenal adenoma may be considered as evidence of some degree of excessive cortisol secretion prior to surgery, these findings collectively suggest that the F-1mgDST cut-off for defining the absence of hypercortisolism in AI patients should be lowered to at least 1.2 µg/dL (33 nmol/L). This idea is supported by previous data showing that F-1mgDST with a cut-off set at about 1.4 µg/dL (39 nmol/L) has the highest accuracy in predicting cardiovascular risk [16, 22] or the incidence of diabetes mellitus [17] in AI patients.

Therefore, our hypothesis is that a subgroup of the so-called “NFAT” patients may still exhibit a hidden hypercortisolism. Therefore, the aim of the present study was to evaluate in a large group of NFAT patients: i) the possible association between metabolic consequences known to be associated with hypercortisolism, such as HT, DM, obesity (OB), dyslipidemia (DL) and CVE, and parameters of hypothalamic–pituitary–adrenal (HPA) axis activity (ACTH and F-1mgDST); ii) the cut-off of the HPA axis activity parameters able to identify NFAT patients with metabolic consequences.

## Patients and methods

### Patients

This is a retrospective cross-sectional observational study. Patients with AI referred to three Italian endocrine centres with high expertise in adrenal disorders were included: Fondazione Cà Granda-Ospedale Maggiore Policlinico in Milan, Istituto Auxologico Italiano IRCCS in Milan, “Casa Sollievo della Sofferenza” Hospital in San Giovanni Rotondo, Foggia. From the institutional databases on adrenal masses, including more than 2500 patients, we recorded the available clinical and biochemical data of all patients with AI and without MACS referred to the participating centres from December 2003 to December 2021.

We selected 615 patients on the basis of the following inclusion and exclusion criteria: (i) referral to the outpatient clinics for adrenal disease of the participating hospitals; (ii) presence of a adrenal mass incidentally found by non-invasive abdominal imaging methods performed for unrelated reasons and confirmed by computed tomography (CT); (iii) F-1mgDST < 1.8 µg/dL, 50 nmol/L; (iv) absence of signs and/or symptoms specific for cortisol excess (moon facies, striae rubrae, skin atrophy, buffalo hump); (v) absence of metastatic diseases; (vi) abdominal CT consistent with the diagnosis of adrenocortical adenomas (i.e.: homogeneous, hypodense below 10 Hounsfield units and with well-shaped features); (vi) absence of drugs or diseases affecting cortisol metabolism and/or secretion (i.e. haematological or rheumatological disorders, gastrointestinal or endocrine diseases, chronic liver or kidney disease, alcoholism, eating disorders, depression) and/or dexamethasone metabolism (i.e. strong CYP3A4 inducers and inhibitors); vii) absence of endocrine alterations consistent with pheochromocytoma or aldosterone-secreting adenoma. In particular, patients with adrenal masses found during the staging for a neoplastic disease or patients with other type of adrenal tumor (about 30% and 10%, respectively, of patients included in the institutional databases) have been excluded. Moreover, in about 20% of patients with NFAT included in the databases, data about comorbidities (DM, HT, OB) were not complete. Among the remaining patients, the 45% was classified as MACS, and therefore, have been excluded.

### Methods

In all patients the following parameters: basal morning (08.00 h) adrenocorticotroph hormone (ACTH) and, in a different day, F-1mgDST levels have been assessed at least once during a baseline evaluation which lasted 1–3 months. For each subject, we reported the baseline mean values of these parameters. The assays used were the same in all centres. Plasma ACTH levels were measured by IRMA (BRAHMS Diagnostica GmbH, Berlin, Germany) and serum cortisol levels were determined by immunoassay (TDX-FLX Abbott, GmbH, Diagnostika kits Wiesbaden-Delkenheim, Germany or by Elecsys Cortisol Immunoassay, Roche Diagnostics, Mannheim, Germany, on CobaS E 602). The coefficients of variation were < 10% for all assays.

We collected the available data regarding age, gender, body mass index (BMI), the presence of HT, DM, DL, OB and CVE. The comorbidities (i.e. HT, DM, DL, OB, CVE) and the biochemical parameters have been assessed at the same time (i.e. baseline evaluation). We defined HT in the presence of systolic blood pressure (SBP) and/or diastolic blood pressure (DBP) > 140 mm Hg and 90 mm Hg, respectively and/or the need for antihypertensive treatment [23] and DM according to the current clinical practice recommendations of the American Diabetes Association [24]. Blood pressure was measured according to the European Society of Cardiology/European Society of Hypertension (ESH/ESC) guidelines. Briefly, the patients sat in a quiet room for several minutes before blood pressure was measured. Blood pressure was measured twice with an interval of 1–2 min between measurements, and further measurements were performed if the first two differed significantly [23]. We defined (DL) in the presence of serum triglyceride levels ≥ 150 mg/dL, total cholesterol levels > 200 mg/dL or high-density lipoprotein cholesterol levels of below 40 mg/dL in men and 50 mg/dL in women. Patients were also considered to have DL if they were receiving an antidyslipidemic drug [25]. We recorded the prevalence of the following CVEs: myocardial infarction, stroke, transient ischemic attack, angina pectoris, pulmonary embolism, intracerebral haemorrhage, peripheral artery disease in the 10 years before the AI was detected. We also recorded data about smoking habits. Subjects were considered current smokers if they smoked ≥ 5 cigarettes/day and/or 10 packs/year [26].

All subjects gave their witnessed informed consent before entering the study, which was approved by local ethical Committees and in accordance with Helsinki Declaration II.

### Study design and statistical analysis

Statistical analysis was performed by SPSS version 28.0 statistical package (IBM Corporation) and GraphPad Prism version 9 (GraphPad Software). Results are expressed as mean ± SD, if not differently specified. The normality of the data distribution was tested using the Kolmogorov–Smirnov test.

The following approach was chosen. First, the receiver operating characteristic (ROC) curve was used to assess the possible association between F-1mgDST and ACTH levels and the prevalent CVE or the presence of either HT or DM or HT *plus* DM or OB or DL. Then, for the statistically significant associations the ROC curve identified the F-1mgDST and/or ACTH cut-off and their associated sensitivities, specificities, as well as areas under the curve (AUC) and it 95% interval of confidence (IC) with the best diagnostic accuracy in individuating NFAT patients with the respective outcomes (i.e. CVE or HT or DM or HT *plus* DM or DL or OB). The Youden’s index (J = sensitivity + specificity − 1) was used to identify the most appropriate cut-off.

Finally, we planned to compare the clinical characteristics of patients subdivided on the basis of the F-1mgDST and/or ACTH cut-off identified by ROC curve. Comparison of continuous variables was performed using Student’s t-test or Mann–Whitney U-test, as appropriate. One-way ANOVA test with post-hoc Tukey multiple comparison test was performed to analyse the differences among the group of patients without comorbidities, the group of patients with HT or DM and the group of patients with HT *plus* DM.

Categorical variables were compared using the χ2 test or Fisher Exact test, as appropriate. Logistic regression analysis was used to assess whether statistically significant associations were independent of possible confounding factors. Patients with missing data about their comorbidities status (HT, DM, DL and OB) were excluded from the study population. Data on CVEs have been obtained from a subgroup of 314 patients and have been separately analyzed.

General Linear Modelling has been used to adjust for potential confounders (i.e. age) in assessing the differences in the parameters of HPA axis secretion between patients with HT and without HT and those with DM and without DM, when needed.

P-values of less than 0.05 were considered significant.

## Results

The results of the ROC curve analysis assessing the associations between F-1mgDST and the explored outcomes (i.e. HT or DM or HT *plus* DM or DL or OB or CVE) are shown in Table 1. The presence of HT, DM and HT *plus* DM, but not of OB, DL and CVE, was significantly associated with F-1mgDST levels. No significant associations were found between these outcomes and ACTH levels (data not shown). The cut-off with the best accuracy in identifying patients with either HT or DM (AUC 0.604, 95% CI 0.560–0.649, sensitivity 60.2%, specificity 56.0%) or HT *plus* DM (AUC 0.611, 95% CI 0.545–0.675, sensitivity 60.4%, specificity 69.6%), was found to be 1.2 µg/dL (33 nmol/L).

**Table 1:** Association between cortisol after dexamethasone suppression test (F-1mgDST) levels and the presence of hypertension, diabetes mellitus, obesity, dyslipidaemia, and cardiovascular events by receiver operating characteristic (ROC) curve

	F-1mgDST (AUC)	p value	95% IC		Youden’s Index		
			Lower limit	Upper limit	Cut-off (µg/dL)	Sensitivity (%)	1-Specificity (%)
Hypertension	**0.588**	**p < 0.001**	**0.543**	**0.633**	**1.18**	**60.5**	**45.5**
Diabetes mellitus	**0.610**	**p < 0.001**	**0.554**	**0.665**	**1.18**	**66.6**	**49.1**
Hypertension plus diabetes mellitus	**0.611**	**p = 0.001**	**0.546**	**0.675**	**1.19**	**60.4**	**49.6**
Obesity	0.507	p = 0.767	0.459	0.556			
Dyslipidaemia	0.494	p = 0.826	0.445	0.544			
Cardiovascular Events**	0.587	p = 0.121	0.484	0.690			

F-1mgDST: cortisol after 1 mg overnight dexamethasone suppression test; AUC: area under the curve; IC: interval of confidence

^*^Data on cardiovascular events (i.e.: myocardial infarction, stroke, transient ischemic attack, angina pectoris, pulmonary embolism, intracerebral hemorrhage, peripheral artery disease) have been obtained from a subgroup of 314 patients

For cortisol multiply × 27.56 to convert from μg/dL to nmol/L. The statistically significant associations are reported in bold

We, then, compared the clinical characteristics of patients with F-1mgDST < 1.2 µg/dL with those of patients with F-1mgDST 1.2–1.79 µg/dL (33–49.4 nmol/L). As shown in Table 2, the latter group showed an older age, lower ACTH levels and a higher prevalence of HT, DM, HT *plus* DM and CVE than the former group.

**Table 2:** Clinical and biochemical features between patients with nonfunctioning adrenal tumors (NFAT) with cortisol after dexamethasone suppression test (F-1mgDST) levels < 1.2 µg/dL and NFAT patients with F-1mgDST levels ≥ 1.2 µg/dL

	All patients (n = 615)	Patients with F-1mgDST levels < 1.2 µg/dL (n = 289)	Patients with F-1mgDST levels ≥ 1.2 µg/dL (n = 326)	P value
Females	289 (47.0)	182 (63.0)	195 (59.8)	0.456
Age (yrs)	**60.15 ± 11.8** **(21.0**–**85.7)**	**57.5 ± 12.3** **(25.0**–**85.7)**	**62.5 ± 10.9** **(21.0**–**85.0)**	**< 0.001**
BMI (kg/m^2^)	28.5 ± 4.9 (18.3–50.0)	28.9 ± 5.2 (18.3–50.0)	28.1 ± 4.6 (18.4–43.3)	0.06
ACTH (pg/mL)	**16.4 ± 11.1** **(1.0**–**60.0)**	**17.7 ± 11.9** **(1.3**–**60.0)**	**15.3 ± 10.1** **(1.0**–**60.0)**	**0.008**
F-1 mg-DST (µg/dL)	**1.17 ± 3.80** **(0.10**–**1.80)**	**0.83 ± 0.22** **(0.10**–**1.10)**	**1.46 ± 0.19** **(1.20**–**1.80)**	**< 0.001**
Size of adenoma (cm)	**2.2 ± 0.9** **(1.0**–**6.0)**	**2.0 ± 0.8** **(1.0**–**6.0)**	**2.4 ± 0.9** **(1.0**–**6.0)**	**< 0.001**
Pts with HT (%)	**281** **(45.7)**	**110** **(38.1)**	**171** **(52.5)**	**< 0.001**
Pts with DM (%)	**114** **(18.5)**	**38** **(13.1)**	**76** **(23.3)**	**0.001**
Pts with HT *plus* DM (%)	**79** **(12.8)**	**24** **(8.3)**	**55** **(16.9)**	**< 0.002**
Pts with OB (%)	212 (34.5)	99 (34.3)	113 (34.7)	0.932
Pts with DL (%)	176 (28.6)	85 (29.4)	91 (27.9)	0.721
Pts with CVE (%)	**33** **(10.5)**	**10** **(3.2)**	**23** **(7.3)**	**0.028**

Pts: patients. F-1mgDST: cortisol after 1 mg overnight dexamethasone suppression test; ACTH: adrenocorticotroph hormone; BMI: body mass index

HT: hypertension; DM: diabetes mellitus; OB: obesity; DL: dyslipidemia; CVE: cardiovascular events (i.e.: myocardial infarction, stroke, transient ischemic attack, angina pectoris, pulmonary embolism, intracerebral hemorrhage, peripheral artery disease). Data on CVE have been obtained from a subgroup of 314 patients

For cortisol multiply × 27.56 to convert from μg/dL to nmol/L. For ACTH multiply × 0.22 to convert from pg/mL to pmol/L

The statistically significant associations are reported in bold

**Fig. 1:**
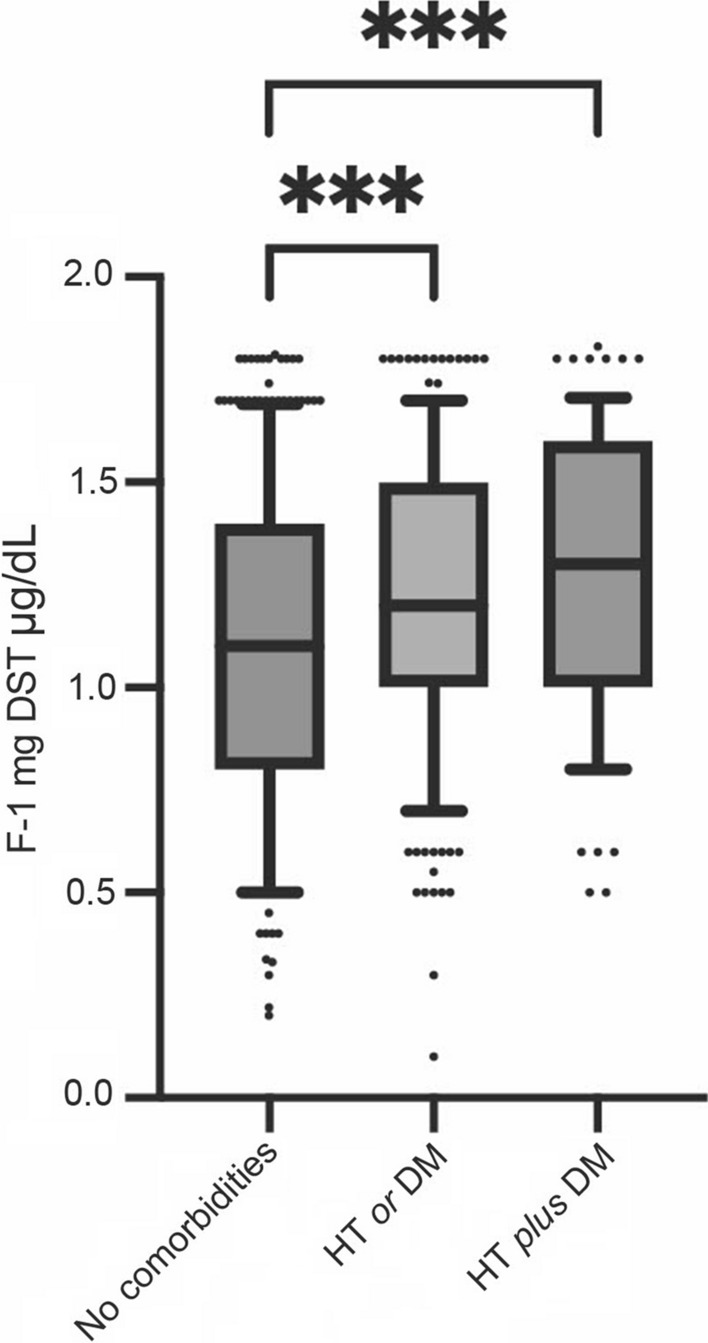
Cortisol after 1 mg overnight dexamethasone suppression test (F-1mgDST) in patients without comorbidities, in patients with hypertension (HT) *or* diabetes (DM) and in patients with HT *plus* DM. Data in the graph are shown as median and interquartile range, the upper and the lower whiskers represent respectively the 90 and the 10 percentiles, *** = p value < 0.001 (One-way ANOVA test with post-hoc Tukey multiple comparison test). F-1mgDST: Cortisol after 1 mg overnight dexamethasone suppression test

Moreover, F-1mgDST values differed among patients with HT *plus* DM, HT *or* DM and patients with no comorbidities. In particular, levels of F-1mgDST were significantly higher in patients with HT (1.23 ± 0.36) as compared with patients without HT (1.11 ± 0.39, p < 0.001) and in patients with DM (1.28 ± 0.34) than in patients without DM (1.14 ± 0.38, p < 0.001), even after adjusting for age (data not shown). Additionally, levels of F-1mgDST were significantly higher in patients with HT *plus* DM (1.29 ± 0.34) and HT *or* DM (1.21 ± 0.36) compared to those without any comorbidity (1.09 ± 0.39), p = 0.0001 and p = 0.0008 respectively (Fig. 1).

Finally, we assessed whether the reported associations between F-1mgDST 1.2–1.79 µg/dL and HT, DM, HT *plus* DM, and CVE were still statistically significant after adjusting for potential confounding factors. As reported in Table 3, F-1mgDST 1.2–1.79 µg/dL was found to be statistically associated with the presence of HT and DM even after adjusting for age, gender, OB, DL and DM (for HT) or HT (for DM), and with the presence of HT *plus* DM even after adjusting for age, gender, OB and DL. At variance, the presence of CVE was only statistically associated with age and not with gender, BMI, active smoking habit and F-1mgDST levels, even though for F-1mgDST levels a tendency toward a statistically significant association was perceivable.

**Table 3:** Independent associations of either hypertension or diabetes mellitus or hypertension *plus* diabetes mellitus or cardiovascular events with F-1mgDST levels ≥ 1.2 µg/dL after adjusting for possible confounding factors

	OR	95% CI	P value
Hypertension			
F-1mgDST > 1.2 µg/dL (yes)	**1.55**	1.08–2.23	**0.018**
Age (1 year increase)	**1.06**	1.04–1.07	**< 0.001**
Gender (female)	1.11	0.77–1.60	0.574
Obesity (yes)	**1.84**	1.26–2.68	**0.002**
Type 2 Diabetes (yes)	**1.81**	1.11–2.96	**0.017**
Dyslipidemia (yes)		‒	
Diabetes			
F-1mgDST > 1.2 µg/dL (yes)	**1.60**	1.01–2.57	**0.045**
Age (1 year increase)	**1.05**	1.02–1.07	**< 0.001**
Gender (female)	**0.57**	0.37–0.90	**0.016**
Obesity (yes)	**2.01**	1.28–3.16	**0.002**
Hypertension (yes)	**1.74**	1.06–2.84	**0.028**
Dyslipidemia (yes)	**2.62**	1.65–4.15	**< 0.001**
Hypertension plus Diabetes			
F-1mgDST > 1.2 µg/dL (yes)	**1.96**	1.12–3.41	**0.018**
Age (1 year increase)	**1.06**	1.03–1.09	**< 0.001**
Gender (female)	0.65	0.38–1.10	0.108
Obesity (yes)	**1.98**	1.17–3.32	**0.010**
Dyslipidemia (yes)	**4.71**	2.78–7.96	**< 0.001**
Cardiovascular events			
F-1mgDST > 1.2 µg/dL (yes)	1.88	0.82–4.28	0.134
Age (1 year increase)	**1.08**	**1.03**–**1.13**	**0.02**
Gender (female)	1.67	0.77–3.62	0.192
BMI (1 kg/m^2^ increase)	1.06	0.98–1.15	0.160
Active smoke (yes)	0.811	0.28–2.40	0.705

OR: odds ratio, 95% CI: 95% Confidence Interval

F-1mgDST: morning cortisol after 1 mg overnight dexamethasone suppression test. BMI: body mass index. Data on cardiovascular events (i.e.: myocardial infarction, stroke, transient ischemic attack, angina pectoris, pulmonary embolism, intracerebral hemorrhage, peripheral artery disease) have been obtained from a subgroup of 314 patients. Subjects were considered current smokers if they smoked ≥ 5 cigarettes/day and/or 10 packs/year [26].The statistically significant associations are reported in bold.

## Discussion

Evidence from the literature suggests that some so-called “NFAT” may still present a partially autonomous cortisol secretion [27]. To date, no data have been available on the possibility that the degree of cortisol secretion in NFAT could be associated with the metabolic consequences known to be related with hypercortisolism, such as HT, DM, OB, DL and CVE. We, therefore, aimed to evaluate in a group of NFAT patients: (i) the possible association between HT, DM, OB, DL and CVE and ACTH and/or F-1mgDST, as estimates of cortisol secretion; (ii) the cut-off values of these parameters to identify NFAT patients with metabolic consequences.

We found that the presence of HT, DM and HT plus DM, but not of OB, DL and CVE was significantly associated with F-1mgDST levels after adjusting for age and comorbidities that could have biased the results. The cut-off with the best accuracy in identifying patients with either HT or DM or HT plus DM was found to be 1.2 µg/dL (33 nmol/L), although the low AUC values suggest a low accuracy of the associations found.

However, the present data could be of interest as they suggest that among AI patients so far diagnosed as not to be affected by hypercortisolism [1], some individuals might, in fact, have a partially autonomous cortisol secretion, which could be reflected in a higher prevalence of cardiovascular and metabolic consequences. These findings are consistent with previous data suggesting that not only in patients with MACS but also in some patients with NFAT, the removal of the adrenal mass could lead to the amelioration of HT and DM [14, 20]. In addition, the incidence of DM was suggested to be increased in patients with NFAT compared with patients without AI [17] and mortality to be similarly increased in NFAT and MACS patients [20]. Finally, the idea that some patients with NFAT may have a partially autonomous cortisol secretion is also reinforced by the notion that a post-surgical condition of hypocortisolism may anyway occur in up to 29% of patients with NFAT who underwent surgery for adenoma size [21, 28]. The lack of association of cortisol secretion with OB and DL is not surprising, as interventional studies suggest that cortisol hypersecretion in MACS has a negative effect particularly on HT and DM, whereas DL and OB are relatively less influenced [14]. At variance, the lack of the association between F-1mgDST and CVE after adjusting for age and other confounders may be due, at least in part, to the reduced sample size compared with the whole number of patients included in the study.

However, the present study also sought to answer the question of whether parameters of HPA axis activity could help in distinguishing NFAT patients with metabolic consequences. We found that ACTH levels were not associated with the presence of comorbidities in NFAT. The finding that ACTH is not a reliable index of the degree of cortisol secretion in AI was somewhat expected since even in patients with MACS, ACTH levels could not be used as the sole parameter of cortisol excess [29], probably because in MACS patients the degree of cortisol hypersecretion may not be sufficient to completely suppress the circadian ACTH secretion [30]. The same issue was therefore predictable to occur also in NFAT patients.

On the other hand, the present data show that a cut-off of F-1mgDST levels set at 1.2 µg/dL (33 nmol/L) is the threshold with the best accuracy in identifying patients with metabolic consequences of a possible cortisol excess. Again, the low AUC levels suggest that this association should be taken cautiously. However, it is of interest that, the same threshold of F-1mgDST was found to be predictive of hypocortisolism after the removal of an adrenal mass with a 100% sensitivity [21]. In general, the idea that the currently used F-1mgDST cut-off of 1.8 µg/dL (50 nmol/L) may not be fully reliable in identifying AI patients with possible hypercortisolism is reinforced by previous data. Indeed, in AI patients the best accuracy for predicting cardiovascular risk and insulin resistance was obtained by using a cut-off of cortisol after two days low dose dexamethasone suppression test set at 1.4 µg/dL (39 nmol/L) and 1.1 µg/dL (30 nmol/L) respectively [16]. Moreover, in a previous study by our group the F-1mgDST cut-off with the best compromise between sensitivity and specificity for predicting cardiovascular events in AI patients was found to be as low as 1.5 µg/dL (41 nmol/L) [22].

However, the potential utility of the F-1mgDST cut-off of 1.2 µg/dL (33 nmol/L) in identifying NFAT patients with metabolic consequences of cortisol excess is clearly debatable, given the low diagnostic accuracy (AUC ~ 0.6 for HT, DM and HT *plus* DM). This is probably because other variables may influence the relation between a slight hypercortisolism and its metabolic consequences in AI patients, such as the production of cortisol precursors with biological effects, the fluctuations in cortisol secretion [27] and the possible interindividual differences in cortisol sensitivity [31, 32].

This study has some limitations. Firstly, its retrospective and cross-sectional design prevents us to draw clear conclusions on the causal effect of cortisol hypersecretion on the outcomes considered (i.e. HT, DM, OB, DL and CVE). Indeed, longitudinal data (although retrospective) would have been of greatest interest, if a long-term follow-up (5 to 10 years) would have been performed. Unfortunately, since these patients were affected with benign NFAT, many of them have been discharged after about 2 years of follow-up, as suggested by the available guidelines [1], preventing us to have reliable long-term longitudinal data. Moreover, although the analyses have been corrected for confounding variables such as age, the age variable still may have an impact on the occurrence of HT and DM. Therefore, the lack of an age- and gender matched control group is a clear limitation of the study. However, given the well-known negative effects of hypercortisolism on these metabolic disorders, a possible causal role of a partial cortisol autonomy may be proposed even on the basis of the present cross-sectional and not-controlled study. Secondly, we do not have data regarding on the circadian rhythm of cortisol, but several data showed a low reliability of midnight salivary cortisol for the diagnosis of MACS and, therefore we do not expect a better performance of this parameter of HPA axis activity in this milder context. In addition, the fact that the available guidelines still suggest that only hypertensive or with unexplainable hypokaliemia AI patients should be screened for hyperaldosteronism [1], prevented us to have data on the aldosterone secretion in many of our patients. Therefore, we cannot exclude that some of these patients were affected by a mild hyperaldosteronism, that could have influenced the cardiometabolic complications.

Thirdly, we do not have data on blood dexamethasone levels to demonstrate the full reliability of F-1mgDST levels as a parameter of cortisol secretion. Finally, we cannot exclude that the high exclusion rate (from about 2500 patients with adrenal masses to 615 NFAT patients) could have determined a selection bias. However, since the main part of patients with adrenal masses has been excluded as affected by neoplastic disease and given, the anyway large sample size, in our opinion, an important confounding effect of the patient selection is unlikely.

Despite these limitations, this is the first study attempting to assess the possible presence of some degree of cortisol hypersecretion (autonomy) in a large sample of patients with what was defined "NFAT" and how to identify it.

In view of the higher cardiometabolic risk in NFAT patients with F-1mgDST 1.2–1.79 µg/dL than in those with F-1mgDST < 1.2 µg/dL (33 nmol/L), it is possible to hypothesize that the term NFAT could be not adequate to represent all AI patients defined so far as having a “non-functioning” adrenal tumour, as already suggested by others [27].

Even though still preliminary, the present data may, therefore, encourage large longitudinal studies aimed to better understand if some NFAT patients may have a relatively increased autonomous secretion of cortisol and/or of its precursors, how to individuate them and how this possible hypersecretion could influence the occurrence of cardio-metabolic complications even in relation to the interindividual difference in cortisol sensitivity.

## Data Availability

The datasets generated and/or analysed during the current study are available in the Zenodo repository, https://doi.org/10.5281/zenodo.7612257.

## Abbreviations

AI: Adrenal incidentalomas
ACTH: Adrenocorticotroph hormone
BMI: Body mass index
CT: Computed tomography
CVE: Cardiovascular events
DM: Diabetes mellitus
DL: Dyslipidemia
F-1mgDST: Cortisol after 1 mg overnight dexamethasone suppression test
HPA: Hypothalamic–pituitary–adrenal
HT: Hypertension
MACS: Mild adrenal cortisol secretion
NFAT: Nonfunctioning adrenal tumors
OB: Obesity
