# Correction: The degree of cortisol secretion is associated with diabetes mellitus and hypertension in patients with nonfunctioning adrenal tumors

**DOI:** 10.1186/s12933-023-01863-y

**Published:** 2023-05-29

**Authors:** Vittoria Favero, Carmen Aresta, Chiara Parazzoli, Elisa Cairoli, Cristina Eller-Vainicher, Serena Palmieri, Antonio Stefano Salcuni, Maura Arosio, Luca Persani, Alfredo Scillitani, Valentina Morelli, Iacopo Chiodini

**Affiliations:** 1 Department of Medical Biotechnology and Translational Medicine, University of Milan, Milan, Italy; 2 Endocrinology Department & Lab of Endocrine and Metabolic Research, IRCCS – Istituto Auxologico Italiano, Milan, Italy; 3 Unit of Endocrinology, Fondazione IRCCS Cà Granda – Ospedale Maggiore Policlinico, Milan, Italy; 4 Unit of Endocrinology and Metabolism, University-Hospital S. Maria Della Misericordia, Udine, Italy; 5 Department of Clinical Sciences and Community Health, University of Milan, Milan, Italy; 6 Ospedale “Casa Sollievo della Sofferenza” IRCCS, San Giovanni Rotondo, FG Italy; 7 Unit of Endocrinology, Ospedale Niguarda Cà Granda, Milan, Italy

Correction to: Cardiovascular Diabetology (2023) 22:102

10.1186/s12933-023-01836-1

Following publication of the original article [1], the author noticed the error in funding section. The correct funding: “The present study has been supported by italian Ministry of Health” instead of “The present study has been partially supported by Istituto Auxologico Italiano (Grant PRECOR Study 2019_01_29_06)”. This has now been corrected with this correction.

The original article has been corrected.
